# A feasibility study using cadaver: Efficacy and safety of the novel automatic urinary catheterization device

**DOI:** 10.1097/MD.0000000000013631

**Published:** 2018-12-21

**Authors:** Seok Kang, Joon Shik Yoon, Chung Ho Lee, Guk-Han Kim, Hyuk Choi, Jae Do Kim, Hong Seok Park

**Affiliations:** a Department of Rehabilitation Medicine, Korea University Guro Hospital, Seoul; b Department of Biomedical Engineering, Eulji University, Seongnam City, Gyeonggi-do; c Department of Medical Science; d Department of Rehabilitation Medicine, Graduate School of Medicine, Korea University; e Department of Urology, Korea University Guro Hospital, Seoul, Republic of Korea.

**Keywords:** automatic device, bladder dysfunction, intermittent cathterization, upper extremity disability

## Abstract

Intermittent catheterization is an effective bladder management strategy for patients with incomplete bladder emptying. For self-catheterization, sufficient hand function in both hands is necessary. We have developed a novel automatic urinary catheterization device to induce self-IC for patients with bladder dysfunction and upper extremity disability. The aim of this study was to investigate the feasibility of this novel automatic catheterization device.

This study was performed using 4 fresh cadavers. First, 400 mL of normal saline was filled into the cadaver bladder. Then, the catheter was inserted using the newly developed device. The catheter insertion was performed 3 times for each cadaver, with the penis positioned at 45°, 90°, and 135°, respectively. A transrectal ultrasonography was performed during the catheterization. We evaluated whether the catheter was successfully inserted into the bladder at each position of penis and whether the urethrovesical junction was injured when inserting the catheter. We also measured the volume of normal saline evacuated from the bladder after successful catheterization.

With the penis positioned at 45° and 90°, catheter insertion was successful without any damage to the urethrovesical junction. However, when the penis was at 135°, the catheter could not be inserted into the bladder. When the automatic catheter insertion was successful, the bladder was successfully emptied. On average, 81.56 ± 3.26% of normal saline was discharged from the bladder and 11.13 ± 2.09% was remained.

The newly developed automatic urinary catheterization device could insert the catheter effectively and safely. This device would be a useful tool for the urinary catheterization of bladder dysfunction patients with upper extremity disability.

## Abstract 2

Supplemental Digital Content is available in the text

## 1 Introduction

Neurogenic bladder dysfunction is one of the most important sequelae after spinal cord injury (SCI), stroke, traumatic brain injury, and multiple sclerosis. In addition, many geriatric patients show voiding difficulty because of dementia, Parkinson syndrome, or benign prostate hypertrophy. Intermittent catheterization (IC) is an effective bladder management strategy for patients with incomplete bladder emptying.^[1]^ IC is particularly crucial for patients with SCI because detrusor-sphincter dyssynergia or high-pressure bladder is associated with life-threatening complications such as upper urinary tract injury and autonomic dysreflexia.^[2–4]^

The IC does not require very specialized skills, but gentle handling is necessary to introduce a catheter through the urethra.^[5]^ For self-catheterization, sufficient hand function in both hands is necessary. In a study by Yilmaz et al, 55.6% of patients with SCI patients who were not able to perform self-IC reported that the reason for their inability to perform self-IC was insufficient hand function.^[6]^

We have developed a novel automatic urinary catheterization device to induce self-IC for patients with bladder dysfunction and upper extremity disability. This device can perform urinary catheterization automatically from sterilization and lubrication to insertion of the catheter into the bladder by simply touching buttons.

The aim of this study was to investigate the feasibility of this novel automatic catheterization device. We have evaluated the efficacy and safety of the device and investigated the safe optimal position of the penis for automatic catheterization.

## 2 Methods

### 2.1 The automatic urinary catheterization device

**Figure 1:**
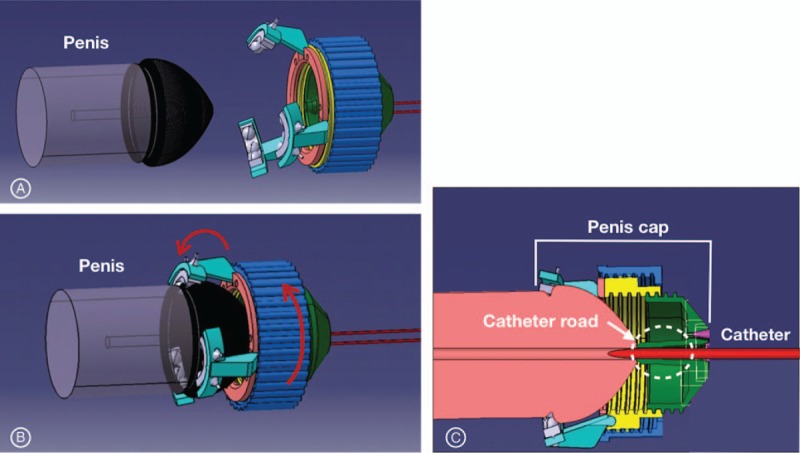
The design of penis cap. (A) The penis cap is separated from the penile glans and the legs on the cap are open. (B) After the penis cap contacting with the glans, rotating the handle on the cap could close the legs and fix the penis. (C) The catheter is inserted into the urethral orifice through the catheter road inside of the penis cap.

**Figure 2:**
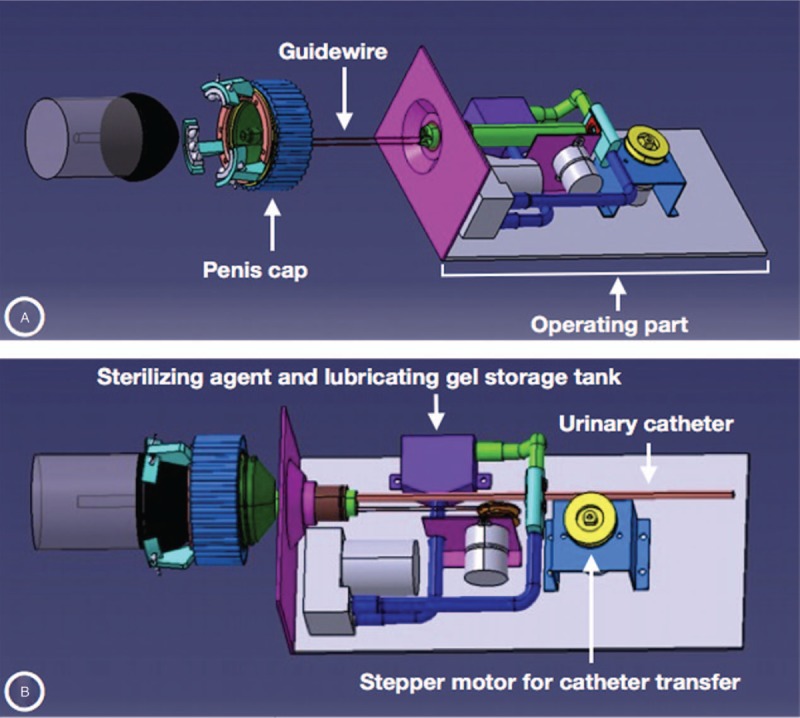
The guidewire system and the internal design of operating part. (A) The guidewire loosened and the operating part is separated from the penis cap. (B) After the operating part is docking with the penis cap, the lubirication and sterilization are conducted as the urinary catheter advanced toward the orifice of the penis cap.

The new automatic urinary catheterization device was designed to enable automatically performing all the process of urinary catheterization, from preparation to execution. The device is composed of 2 parts: penis cap and operating part (Figs. 1 and 2). The 2 parts are connected with a guide wire. The function of penis cap is holding the glans of penis and guiding the operating part to the orifice of penis. In the operating part, storage tank of lubricant and sterile agent, urethral catheter, and stepper motor for the transfer are contained. The functions of operating part are sterilization and lubrication of the catheter, transfer of the device to penis cap in guidance of guide wire, and advance of urinary catheter into the urethral orifice.

The 1st step of the urinary catheterization is holding the penis and correctly targeting the orifice. In this device, the penis cap and guide wire were used to target the urethral orifice correctly (Figs. 1 and 2A). In the penis cap, there is a small protrusion (catheter road in Fig. 1C) with a hole extending from the opening for the catheter path. It could fit with the orifice of urethra and guide the catheter into the orifice. When wearing the penis cap on the glans of penis, the catheter road should fit into the external orifice of penis and a catheter could be inserted into the urethral orifice through it. The next step is the sterilization and lubrication of the catheter before insertion into the urethral orifice. The sterilizing agent and lubricating gel could be stored in the operating part of the device (Fig. 2B). When performing the catheterization, the catheter could be coated with the sterilizing agent and lubrication gel. The final step is the insertion of the catheter. The operating part was designed to transfer and insert the catheter using stepper motor after docking with the penis cap (Fig. 2B).

**Figure 3:**
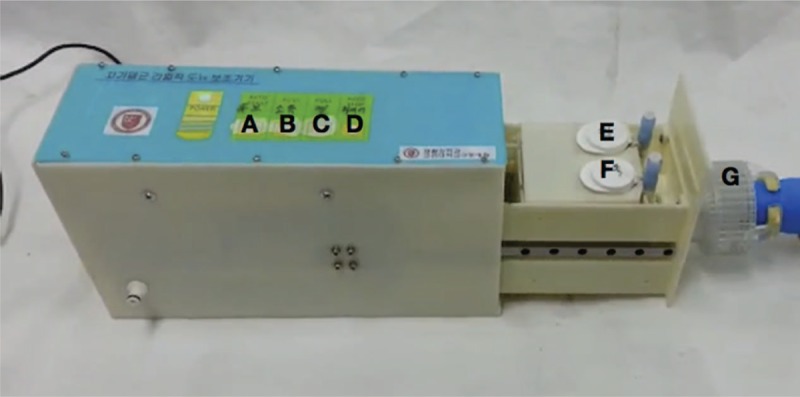
A prototype of the novel automatic urinary catheterization device. (A) Catheter progress and retraction button. (B) Sterilization button. (C) Lubrication button. (D) Start and finish button. (E) Chamber for lubricant. (F) Chamber for antiseptic agent. (G) Penis cap.

A prototype device for automatic urinary catheterization was made for this study (Fig. 3 and Suppl. 1). There were 4 touch buttons on the prototype device, which are catheter progress and retraction button, sterilization button, lubrication button, and start and finish buttons. In the operating part of the prototype device, the chambers for antiseptic agent and lubricant were located. The catheterization process could be started by touching the insertion button. When the bladder emptying is finished or if the catheter insertion is failed, the device could retract the catheter in response to touching a retraction button.

### 2.2 Protocol

**Figure 4:**
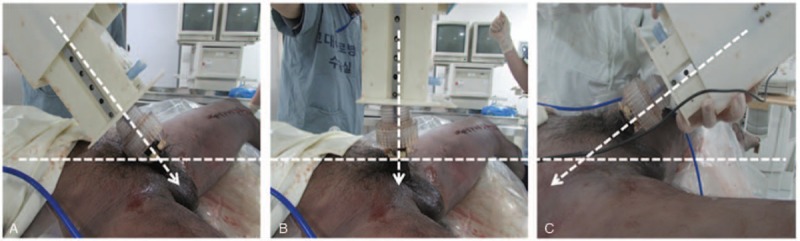
The penis angle of catheter insertion. (A) 45°, (B) 90°, (C) 135° to the abdomen.

This is an experiment for testing the feasibility of new automatic urinary catheterization device. This study was exempt from approval by the Institutional Review Board of the Korea University Guro Hospital because it was a research using cadavers. Four fresh male cadavers were used for this study. First, the bladders of cadavers were filled with 400 mL of normal saline through a manually inserted urinary catheter. A bladder scanner (BioCon-700; Mcube Technology, Seoul, Korea) was used to confirm that the bladders were filled with the saline. Transrectal ultrasonography (TRUS) was performed to investigate any urethrovesical junction injury using an endorectal C9-5 transducer of a high-resolution ultrasonography (Philips Ultrasound Inc, Bothell, WA). Then, a 16 Fr catheter was inserted using the newly developed device. The catheterizations were performed 3 times for each cadaver to investigate the optimal penile position. The penis was positioned at angles of 45°, 90°, and 135° to the abdomen, respectively (Fig. 4). TRUS was performed during the catheterization to confirm that the catheter was inserted into the bladder without injury of the urethrovesical junction. To evaluate effective evacuation, the volume of normal saline discharged through the catheter was measured and the residual volume was evaluated using a bladder scanner. We compressed the bladder during evacuation because the bladders of cadavers did not have contractile forces.

## 3 Results

**Figure 5:**
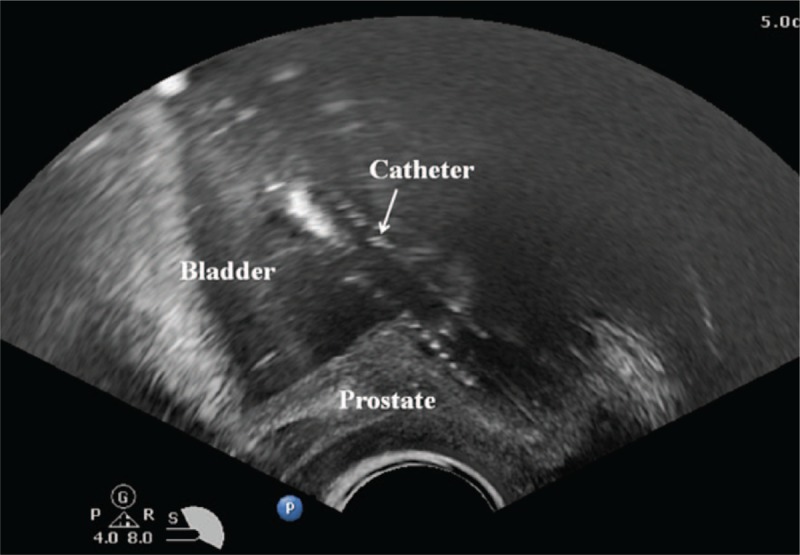
The ultrasonographic findings of urethrovesical junction after successful catheter insertion.

During the catheterization using the newly developed automatic device, the catheter could not be inserted when the penis was positioned at an angle of 135° in all the cadavers. However, the catheterization was smoothly done when the penis was positioned at an angle of 90° or 45° (Fig. 5 and Suppl. 2). The anatomic structures around the urethrovesical junction were not injured during the catheterization. Bladder evacuation was successfully performed after the automatic catheter was inserted into the bladder. The mean volume of discharged normal saline was 326.25 ± 13.02 mL (81.56 ± 3.26%) and the residue was 44.50 ± 8.35 mL (11.13 ± 2.09%).

## 4 Discussion

This study has shown that the newly developed automatic urinary catheterization device can insert a urinary catheter into the male urethra safely and induce bladder emptying effectively. Automatic catheterization can be successfully performed with a penile angle of <90° to the abdomen.

Although the simplest method for managing bladder emptying dysfunction is placement of an indwelling catheter, the chronic use of an indwelling catheter is associated with severe complications, such as urinary tract infection, development of urinary stones, and vesicoureteral reflux.^[7]^ IC is superior to any other bladder managing methods for bladder emptying, without causing any complications.^[7]^ In 1 study, the incidence rate of urinary tract infection was not significantly different between self normal voiding and IC, while the other bladder emptying methods, including indwelling catheters and the crede maneuver, showed a significantly higher rate of UTI.^[8]^ Furthermore, self-IC has advantages in maintaining a positive body image and improving the quality of life.^[9,10]^ Thus, IC is widely accepted as a much safer bladder management method.

However, many individuals with neurogenic bladder, especially patients with SCI, are unable to perform self-IC and have to continue management with indwelling catheters. Despite the development of several assistive devices to assist with IC,^[10,11]^ most of patients with higher cervical cord injury cannot perform self-IC. Kriz et al reported that no patients with SCI at the C4 level, 36.6% at the C5 level, 66.7% at C6 level, and 85.7% at the C7 level could perform independent self-IC.^[4]^ Inadequate upper extremity function is the main obstacle to self-IC.^[6]^

As the novel device used in the present study can perform urinary catheterization automatically by only touching a button, even patients with a high cervical level SCI can perform self-IC with this device. Because IC is one of the most important daily living activities for patients with SCI, this novel device could be an effective tool for increasing their independence in daily living.

When performing manual urinary catheterization in a male patient, the penis should be stretched forward and held in an upward direction because there are several interrupting structures such as the prostate and the urethrovesical angle at the external sphincter. In the present study, for automatic catheterization the penis should also be positioned in an angle of <90° to the abdomen. Since most patients with neurogenic bladder are wheelchair bound, IC is generally performed in a seated or recumbent posture and the penis can be placed at an angle of 90° or less. The novel device in the present study showed safe and effective catheterization at a penile angle of 90° and 45°. Thus, automatic catheterization would be possible in a seated or recumbent posture.

The purpose of this device was to encourage a self-catheterization in bladder dysfunction patients with upper extremity disability. However, the automatic urinary catheterization using this device could cause lesser pain than the manual procedure, because of constant insertion velocity, smooth movement of catheter, and uniform coating of lubricant. Thus, this device would have a practical value for the purpose without pain and discomfort in urinary catheterization.

The followings are the precautions and complications that could occur while using this device in practice. First, although the catheter was inserted into the bladder without any injury to surrounding tissues in cadaver study, the automatic catheterization could cause unintentional injury to surrounding tissues in a real clinical environment. Particular attention should be paid to the urethral injury. Structures around the bladder neck, such as the prostate, are also susceptible to damage. Patients with high-level SCI require caution because pain during catheter insertion could lead to an emergency such as autonomic dysreflexia. In addition, urinary tract infections due to contamination of catheter may occur. These precautions or complications are not very different from those requiring in manual catheter insertion. However, when performing the automatic catheterization, particular attention should be paid about serious complication of a penile rupture during the procedure, because unintentional erection could occur in patients with spinal cord injuries.

### 4.1 Limitations

The most critical limitation is that this novel device was developed for male patients only. In further research, development of a device for female patients should be pursued. Secondly, the prototype device is large and unacceptable for practical use. Thus, we consider downsizing the device to be the main objective in follow-up research. In addition, it could be one of the most critical limitations that the device could not fit all the differences of physical dimensions of the body. To overcome this limitation, we had separated the device into 2 parts, penis cap and operating part. We think that the differences of physical dimensions could be fitted by using diverse sizes of penis cap. Furthermore, as the urethral orifice is not centered on the penis, it would be necessary to modify the penis cap to be more anatomically suitable. In the future development, more various types and sizes of penis caps should be developed and evaluated for the practical application.

## 5 Conclusion

The newly developed automatic urinary catheterization device can insert a catheter effectively and safely. This device can be a useful tool for urinary catheterization in bladder emptying disorder patients with upper extremity disability.

## Author contributions

**Conceptualization:** Seok Kang, Joon Shik Yoon, Hyuk Choi, Jae Do Kim, Hong Seok Park.

**Data curation:** Seok Kang.

**Formal analysis:** Seok Kang.

**Funding acquisition:** Joon Shik Yoon.

**Investigation:** Seok Kang, Joon Shik Yoon, Chung Ho Lee, Guk-Han Kim, Hyuk Choi, Hong Seok Park.

**Methodology:** Seok Kang, Guk-Han Kim, Hyuk Choi, Hong Seok Park.

**Resources:** Guk-Han Kim, Hong Seok Park.

**Validation:** Hong Seok Park.

**Writing – original draft:** Seok Kang.

**Writing – review & editing:** Seok Kang.

## Supplementary Material

Supplemental Digital Content

## Supplementary Material

Supplemental Digital Content
